# A promising case of preclinical-clinical translation: β-adrenoceptor blockade from the oxygen-induced retinopathy model to retinopathy of prematurity

**DOI:** 10.3389/fphys.2024.1408605

**Published:** 2024-06-13

**Authors:** Maurizio Cammalleri, Luca Filippi, Massimo Dal Monte, Paola Bagnoli

**Affiliations:** ^1^ Department of Biology, University of Pisa, Pisa, Italy; ^2^ Neonatology Unit, Azienda Ospedaliero-Universitaria Pisana, Pisa, Italy; ^3^ Department of Clinical and Experimental Medicine, University of Pisa, Pisa, Italy

**Keywords:** hypoxia, retinal angiogenesis, anti-VEGF therapy, alternative strategies, translational failure, sympathetic overstimulation

## Abstract

Although compartmentalization of the eye seems to promote its experimental manipulation, drug penetration to its posterior part is severely limited by hard barriers thus hindering drug development for eye diseases. In particular, angiogenesis-related retinal diseases share common mechanisms and are responsible for the majority of cases of blindness. Their prevalence is globally increasing mostly because of the increased incidence of systemic pathologies in the adult. Despite the number of preclinical findings demonstrating the efficacy of novel treatments, therapy of retinal neovascular diseases still remains confined to intravitreal anti-vascular endothelial growth factor treatments with some extension to anti-inflammatory therapy. In the *mare magnum* of preclinical findings aimed to develop novel avenues for future therapies, most compounds, despite their efficacy in experimental models, do not seem to meet the criteria for their therapeutic application. In particular, the groove between preclinical findings and their clinical application increases instead of decreasing and the attempt to bridging the gap between them creates intense frustration and a sense of defeat. In this complex scenario, we will discuss here the role that overactivation of the sympathetic system plays in retinal vessel proliferation in response to hypoxia using the oxygen-induced retinopathy (OIR) model. The potential application of the beta-adrenoceptor (β-AR) blockade with propranolol to the treatment of retinopathy of prematurity will be also discussed in light of preclinical findings in the OIR model and clinical trials using propranolol in preterm infants either *per os* or as eye drops.

## Introduction

**FIGURE 1:**
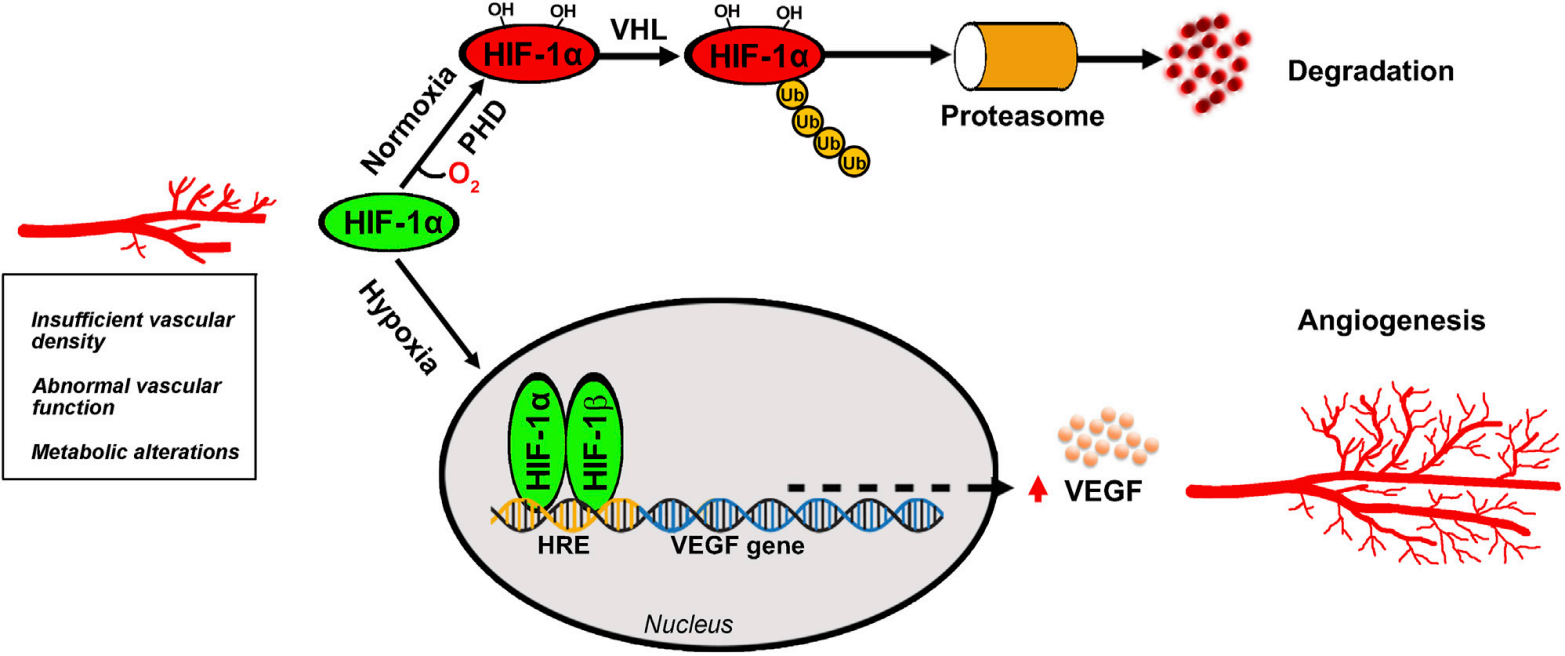
Angiogenic process in the retina. Oxygen deficiency at the retinal tissue prevents HIF-1α hydroxylation by PHD thus escaping VHL-induced proteasomal degradation and leading to HIF-1α dimerization with HIF-1β that generates the active form of HIF-1. After its translocation into the nucleus, HIF-1 binds to HRE to then promoting the transcription of several target genes of which the VEGF gene leads to the production of VEGF protein that binds to its receptors to trigger the angiogenic cascade culminating in new vessel proliferation. HIF-1, hypoxia-inducible factor-1; HRE, hypoxia response element; PHD, prolyl hydroxylase domain protein; VEGF, vascular endothelial growth factor; VHL, Von Hippel-Lindau.

Higher mammals relay on sight that has evolved into one of the most important senses to allow rapid detection of environmental perturbations and to enable species survival. Focusing light onto the retina causes chemical changes in the photosensitive cells thus allowing to trigger a cascade of events leading to the generation of nerve impulses that travel to the brain to support visual perception. The high energy demand of the retina is normally matched with a large supply of oxygen and nutrients through a well-organized vascular system that also participates to remove waste products. Adequate blood supply maintains the vitality of the retina and ensures a correct visual function while altered retinal vasculature poses significant threats to sight in affected individuals. When metabolic demands by the retina exceed its oxygen supply, then compensatory mechanisms are put in place to preserve oxygen delivery. As the retina lacks oxygen reserve, its vessels undergo remodeling and proliferation in response to pathological hypoxia. The cascade of events leading to retinal vessel proliferation or angiogenesis is driven by the hypoxia inducible factors (HIFs) that are regulated by enzymes, the HIF-prolyl hydroxylase domain proteins, which in the presence of low oxygen lose their hydroxylation activity. Thus, HIF-1α escapes from degradation and enters the nucleus to dimerize with HIF-1β to, then, trigger a cascade of events leading to the transcription of a number of proangiogenic genes. Among them, the vascular endothelial growth factor (VEGF) gene plays a major role to increase retinal vessel density in order to guarantee correct oxygen delivery to sustain the metabolic demand by the tissue (Figure 1).

Abnormal vascular proliferation consists of fine, superficial vessels that are often associated with altered interendothelial cell junctions leading to dysfunctional blood retinal barrier (BRB) whose integrity is essential to maintain retinal structure and function. Increased vascular permeability consequent to BRB breakdown causes leaky blood vessels leading to abnormal accumulation of fluid in the subretinal space, a major sign of macular edema.

### Angiogenesis-associated retinal diseases

Hypoxia-induced retinal vessel growth characterizes several pathological and degenerative diseases of the retina among which retinopathy of prematurity (ROP) is characterized by abnormal vascularization as a consequence of preterm birth (Pascarella et al., 2024). Among neovascular diseases of the adult, proliferative diabetic retinopathy (PDR) is the more advanced stage of DR that is characterized by thin and weak blood vessels whose bleeding can cause scar tissue leading to retinal detachment (Kaur et al., 2024). In addition, neovascular age-related macular degeneration (nAMD) involves abnormal proliferation of choroidal vessels, which enter into the subretinal space through the Bruch’s membrane. In this respect, fluid extravasation caused by choroidal neovascularization (CNV) produces severe damage to the retina leading to retinal cell death (Salas et al., 2023). AMD is still the most prevalent diagnosis followed by PDR that shares common pathophysiology including altered angiogenic balance and oxidative stress-induced inflammation. In the case of PDR, hyperglicemia results in oxidative stress and inflammation leading to microvascular cell death (González et al., 2023). Outcoming retinal ischemia triggers upregulation of VEGF expression and downregulation of anti-angiogenic factors that are potent inhibitors of the angiogenic response. In physiological conditions, anti-angiogenic factors can block retinal vessel proliferation until, on certain conditions, the pro-angiogenic regulators predominate and the endothelium starts to proliferate. Among anti-angiogenic peptides, pigment epithelium-derived factor, a multifunctional secreted protein that is involved in the balance between pro-angiogenic and anti-angiogenic environment by interacting with the extracellular matrix (Brook et al., 2019).

Of the proliferative retinopathies, ROP, although less prevalent, has attracted much attention because of its increasing incidence rate among premature infants with low body weight. In USA, for instance, ROP increased from 4.4% in 2003 to 8.1% in 2019 (86% relative increase) nationwide with the largest increase found in Black (5.8%–11.6%) and Hispanic infants (4.6%–8.2%) (Ying and Quinn, 2023). In this respect, ROP is a leading cause of blindness in children worldwide due to increasing survival rates of premature infants also in developing countries because of the improvement in the nursing care of high-risk newborn babies. Finally, ROP modeling by the oxygen-induced retinopathy (OIR) model represents a fundamental approach for studying the pathophysiology of angiogenesis-related retinal diseases and the effects of novel treatments (Gammons and Bates, 2016). The OIR model is largely recognized as a well accessible model of retinal vessel proliferation that allows an easy quantification of abnormal neovessels together with the possibility to perform electrophysiological, histological and molecular analyses (Connor et al., 2009).

### Therapeutics for angiogenesis-related retinal diseases

Understanding the mechanisms governing retinal vessel proliferation in pathological condition has allowed to develop preventive and interventional therapeutics for angiogenesis-related retinal diseases although the fragility of the retinal tissue and the presence of the BRB limiting the transport of pharmacological agents has proved problematic in the therapeutic regulation of angiogenesis. Since the late 20th, laser photocoagulation was used to destroy the part of the retina that lacks oxygen in order to avoid neovessel proliferation and to prevent hemorrhages in the vitreous, while vitrectomy was applied when blood extravasation affects visual acuity (Oellers and Mahmoud, 2016; Zhang et al., 2023). In this respect, panretinal photocoagulation is still the main treatment for PDR, allowing the regression of retinal and papillary new vessels (Raman et al., 2022).

We had to wait until early 21st century for pharmacological treatment of angiogenesis-associated eye syndromes through the repurposing of anti-VEGF therapies against the majority of eye diseases associated to VEGF-related vessel proliferation (Bahr and Bakri, 2023; Hang et al., 2023). However, some concerns have been raised regarding the use of anti-VEGF agents particularly when the disease management still needs intravitreal administration despite the enormous effort to investigate novel ocular delivery that guarantees an appropriate permeation. Repeated injections may cause several complications including endophthalmitis, which is characterized by a persistent increase of intraocular pressure (Cox et al., 2021). In this respect, anti-VEGF agents, which, through an extended release, may minimize the number of intravitreal administrations, are under development (Xu et al., 2022). Additional effort is also aimed at fighting anti-VEGF resistance to the repeated administration of standard treatments over time. In this respect, combining anti-VEGF therapy with intervening on alternative pathways that could potentially bypass development of resistance to anti-VEGF treatment should help to discover new therapeutic strategies for overcoming anti-VEGF resistance (Sharma et al., 2023). For instance, in the vitreous fluid of patients suffering from neovascular eye disorders, VEGF may be only one of the detectable proangiogenic factors (Simó et al., 2006), which may cause resistance to anti-VEGF therapies. In this respect, novel anti-angiogenic molecules with increased intravitreal residence time to produce long-lasting effects are under development (Martinez-Alejo et al., 2022).

A lot of pressure is also exerted towards the development of molecules that, administered through a non-invasive route, should normalize instead of abnegating retinal VEGF levels. In fact, VEGF has been shown to act as a trophic factor in the retina by promoting retinal cell survival (Nishijima et al., 2007). In the case of retinal ganglion cells (RGCs), for instance, autocrine role of VEGF in sustaining their own growth and survival is in line with the clinical observation that glaucomatous patients injected with anti-VEGF because of AMD or DME comorbidity, display a significant RGC axon loss (Froger et al., 2020). Finally, the area of targeting VEGF receptors progresses with novel approaches and therapeutically based hope for best clinical outcomes for patients through developing anti-angiogenic therapies (Jiang et al., 2023). Beside anti-VEGF therapy, targeting inflammatory processes and preventing Müller cell gliosis, a major sign of retinal inflammation, represents the rationale for intra-ocular steroid implants (Abdulla et al., 2022) although the increasing multitude of novel inflammatory targets as recognized by preclinical findings may provide confounding contribution to the exploitation of novel treatments.

### Limited investment of preclinical findings

Anti-VEGF therapies although successfully counteracting neovascularization progression are facing several issues that may, at least in part, justify the increasing need of preclinical findings aimed to develop alternative treatments. Therefore, the literature churns out at a frenetic pace preclinical findings on novel treatments for angiogenesis-associated retinal diseases.

Despite the efforts of basic research to produce preclinical data of high success rate, the groove between preclinical findings and their clinical application increases instead of decreasing. In particular, treatments that are effective in experimental models do not appear to translate to clinical efficacy. Several fundamental problems may explain the translational failures including the lack of information on the pathogenesis of a given disease and the fact that no single animal model completely covers the complexity of the human disease (Frangogiannis, 2022).

When considering the overall success rate of clinical drug development, most of the drug candidates potentially validated at the preclinical stage have not yet made their way to the clinic with a failure rate higher than 90% (Sun et al., 2022). In the case of novel drugs for neovascular diseases of the retina, those that received approval and entered the market still remain confined to their intraocular delivery. However, advancement in ocular drug delivery and the pharmacokinetic of novel drugs may improve the drug efficacy and reduce the treatment burden (Kim and Woo, 2021). In this respect, the cost of developing drugs that do reach the market can be estimated up to three billion USD on average (Wouters et al., 2020) on a global ophthalmic drug market size expected to reach about USD 66 billion by 2030 according to Straits Research (2024). If one considers the large amount of data produced by preclinical studies, the difficulty to fulfill clinical needs is an extremely frustrating situation.

Validation of target assessment includes numerous steps starting from preclinical animal studies that display several limitations in their experimental design and statistical analysis (Vofo and Chowers, 2023). To narrow the field, two major prerequisites have to be taken into consideration when addressing a molecule with potential therapeutic application in angiogenesis-related retinal diseases. The first would be to target angiogenic regulators other than VEGF although escaping VEGF-targeting approaches does not automatically mean to offer a better alternative to the current anti-VEGF therapy. The second would be to identify hypothetical drugs to be delivered by alternative routes, thus avoiding the potential side effects of intraocular administration. In addition, the therapeutic approach is strongly complicated by the need to target multiple pathogenic pathways also depending on the disease stage. In the case of AMD, for instance, intervening on early or intermediate stage with targeted therapies may help to halt or delay disease progression (Khachigian et al., 2023; Lad et al., 2023).

The reliability of preclinical studies is further complicated by animal modeling of angiogenesis-associated retinal diseases that not always mimic the human disease. As to PDR, for instance, the modeling situation is much complicated by the fact that in humans, PDR takes years to be diagnosed while animal models do not include the late proliferative stage due to their short lifespan and thus the shorter duration of the disease (Quiroz and Yazdanyar, 2021). In contrast, the rodent OIR model reliably mimics hypoxia-driven angiogenesis and is widely used to replicate ischemic retinopathies (Vähätupa et al., 2020). Drug development is additionally complicated by the confounding factor addressed by the large number of studies demonstrating the efficacy of diet supplements with antioxidant and anti-inflammatory properties as oxidative stress and inflammation are severely implicated in angiogenesis-associated retinal diseases (Rusciano and Bagnoli, 2023). Although preclinical findings are extremely promising for anti-angiogenic efficacy of diet supplements, major obstacles to their use in clinical settings include their poor bioavailability as they would require upper intake levels to replace their fast elimination. In addition, the difficulty of designing clinical studies that take into consideration the complex nature of the supplements and the clinically relevant endpoints further complicates their transfer to clinical trials. Finally, the effective dosage used in animal models significantly limits the translation of diet supplements to humans. In fact, doses used in rodent models are generally much higher than the maximal amount compatible with a diet supplement in humans because of the basal metabolic rate in rodents, which is almost 6 times faster than in humans (Reagan-Shaw et al., 2008). The additional fact that mimicking a retinal disease in rodents requires weeks as compared to decades in humans makes the need of much higher doses to demonstrate the efficacy of certain molecules in a limited lifespan.

Another major problem is that when combined with therapies, dietary supplements contain ingredients that have strong biological effects which may conflict with a given drug or the medical condition for which the drug has been prescribed (Féart, 2020). Beside their interaction with prescribed drugs, the greatest concern with using dietary supplements is that products may be laced by hidden drugs either unlabeled or sometimes falsely marketed as dietary supplements limiting their safety before reaching the consumer (Food and Drug Administration, 2021).

Among futuristic treatments of neovascular diseases of the retina, inhibiting transcription factors that bind the VEGF promoter to initiate and activate VEGF gene transcription, mRNA-based therapeutics for the production of any protein/peptide by utilizing the protein synthesis process in transfected cells or gene therapy to block the *in situ* production of VEGF or to target sick genes, although having a lot of potential still needs additional experimental demonstration of safety and efficacy (Lee D. et al., 2022; Sobh et al., 2023). On the other hand, the identification of additional disease-related genes, their feasibility at the clinical level and prevention of potential immune responses elicited by gene delivery systems may further expand retina’s armamentarium using the latest treatments for nAMD and DME. In addition to gene therapy, cell-based therapies including the application of perinatal derivatives to restore or replace the damaged retinal vasculature and the retinal neurons that are damaged and/or degenerating from the hypoxic insult although having a lot of potential still need preclinical work to identify additional disease-related genes, to improve targeting of the delivery systems, to prevent potential immune responses, to evaluate the clinical trial feasibility process, etc. (Hinkle et al., 2021).

### Among promising therapies: the case of β-AR blockade

The OIR model, in mouse pups, mimics retinal vessel proliferation in response to hypoxia as it occurs in preterm infants with ROP, which is defined as a vasoproliferative disorder affecting the immature retina. In the vascularization process, retinal vessel proliferation is triggered by VEGF released in response to *in utero* hypoxia. After preterm birth, however, vessel growth slows or stops because of the relative hyperoxia that leaves the peripheral retina in an avascular condition leading to hypoxia. Hypoxia then triggers the proangiogenic cascade leading to aberrant vascular growth, including the formation of fibrous scars with the potential complication of retinal detachment. In that case, laser photocoagulation is utilized to arrest vascular growth although anti-VEGF drugs have emerged as treatment options beside the outstanding questions regarding their efficacy (Nowroozzadeh et al., 2023). In fact, in the last few years, anti-VEGF agents are being used as monotherapy or as coadjuvant with laser photocoagulation, which, on the other hand, may destroy parts of the retina thus leading to significant eye complications later in life.

Increasing incidence of neovascular retinal diseases including ROP has aroused much interest in the development of novel therapies as also determined by the increased number of preclinical studies using the OIR model with almost 29.100 published papers from 2020 to 2023 in respect to almost 18.600 from 2016 to 2019 with an increase of about 55% (results retrieved from Google Scholar). On the other hand, the OIR model covers an extended field of investigations including those aimed at developing novel therapies to treat abnormal leaky neovessels as a specific feature of pathological angiogenesis.

In search for novel therapies to inhibit ischemic retinopathy, the possibility to counteract retinal vessel proliferation by blocking β-ARs results from the fact that mechanisms governing compensatory angiogenesis in the retina include a positive feedback circuit comprising hypoxia-driven chemosensor stimulation that, in turn, may directly increase sympathetic outflow leading to norepinephrine (NE) release to the eye, which overactivates β-ARs in the retina. In this respect, in rodent models, the increase in plasma NE after asphyxia has been associated with nerve ending release of NE mainly due to hypoxia that stimulates the sympathetic system (Borovsky et al., 1998).

Around the early 2010, several findings prompted us to use the OIR model in order to investigate the efficacy of the β-AR blocker propranolol to counteract angiogenic processes. The principal reason for candidacy of propranolol was the accidental discovery of its anti-angiogenic efficacy in infantile hemangioma (IH), the most common vascular tumor in low-birth-weight infants in which it is often associated with ROP (Léauté-Labrèze et al., 2008). The concomitant finding that treatment with anti-hypertensive β-blockers reduces disease progression in patients with melanoma presumably by reducing tumor angiogenesis (De Giorgi et al., 2011) further supported a potential anti-angiogenic role of propranolol in the OIR model.

After propranolol application for the treatment of tachycardia and hypertension in the 1960s, its repurposing to counteract rare vascular diseases has allowed a quick and less expensive alternative with an added value of its immediate use in clinical trials because its well-known safety profile (Cuesta, et al., 2022). Additional advantage is the lipophilic nature of propranolol that allows it to cross the blood-brain barrier and the BRB, on the one hand facilitating its availability after systemic administration, but on the other enhancing the risk of side effects.

Propranolol is a non-selective competitive antagonist that blocks β-AR binding to NE thus inhibiting sympathetic activity. Catecholamines work through the formation of a hydrogen bond between their hydroxyl group and the receptors. However, propranolol prevents the link between NE and β-ARs but does not activate the receptors by lacking the hydroxyl group that allows β-AR activation. β-AR blocking activity is mediated by the S(−) enantiomer while the R(+) enantiomer has lower affinity for adrenergic receptors. It rather appears to counteract neovessel proliferation by entering the cell and interfering with the dimerization of the transcription factor Sex determining Region Y Box 18 (SOX18) that acts as a master regulator of neovessel formation (Seebauer et al., 2022).

Propranolol binds to both β1-and β2-ARs and induces antagonizing effects via both receptors while selective blockers like atenolol bind to the β1-ARs. β1-AR plays a critical role in maintaining blood pressure homeostasis and cardiac output and its antagonism is widely used in the treatment of hypertension, heart failure and ischemic heart disease (Motiejunaite et al., 2021). β2-ARs are predominantly present in airway smooth muscles and their agonism is clinically taken advantage in the management of bronchospasm as in patients with bronchial asthma and chronic obstructive pulmonary disease (Yawn et al., 2021).

In the OIR model, propranolol has been found to effectively inhibit the hypoxia-induced increase in HIF-1α and VEGF expression and the consecutive neovascular response suggesting that β1-/β2-AR blockade is protective against hypoxia-induced angiogenesis (Casini et al., 2014). In respect to anti-VEGF treatment, however, propranolol recovers control levels of VEGF without knocking them down, a harmful event for retinal health. Additionally, propranolol has been shown to ameliorate the hypoxia-associated breakdown of the BRB, which is known to maintain the integrity of the retinal microenvironment. BRB breakdown, in contrast, may result in increased fluid accumulation that significantly contributes to neovascular pathologies of the retina (O'Leary and Campbell, 2023). The anti-angiogenic efficacy of propranolol is mimicked by the β2-AR specific antagonist ICI-118,551, but not by the β1-AR blocker atenolol suggesting that β2-AR plays a major role in the angiogenic response to hypoxia (Martini et al., 2011). Because of the lack of FDA-approved selective β2-AR antagonists, propranolol role has continued to be investigated in the OIR model for its potential translation ability.

### β2-AR coupling to VEGF

The pharmacological characteristics of propranolol are much in line with the possibility that it may counteract retinal angiogenesis associated to β2-AR overactivity. Indeed, β2-ARs may play a role in the angiogenic response to hypoxia through their regulation of the VEGF cascade. For instance, strong β2-AR expression in metastases has been associated with clinical benefit of anti-VEGF treatment in reducing tumor angiogenesis as it occurs in patients with melanoma (Schuster et al., 2023). At the preclinical level, in human endothelial cells, β2-AR agonism or β2-AR overexpression in response to hypoxia lead to endothelial cell proliferation, migration and tube formation while β2-AR deletion inhibits VEGF expression and tube formation (Xanthopoulos et al., 2021).

Molecular mechanisms triggered by β2-AR activation converge onto VEGF accumulation through a complex network of intracellular effectors that combine with each other to produce cellular and tissue responses. Unravelling signaling networks downstream β2-ARs has important implications for better understanding of their role in angiogenesis and might outline further novel therapeutic strategies.

Described below is the signaling crosstalk activated by β2-AR that has been detailed as much as possible because of the complex intermingling of the different pathways coupled to its activation. The signaling pathway of β2-AR starts after its activation by an agonist that leads the alpha subunit of the Gs protein to detach and reattach to adenylate cyclase, thus converting adenosine triphosphate into cyclic adenosine monophosphate (cAMP). cAMP acts as a second messenger by binding to the regulatory subunits of protein kinase A (PKA) to release the catalytic subunits, which in turn phosphorylate a number of intracellular proteins that converge on HIF-1 transcriptional activity leading to VEGF production. For instance, in a mouse model of pancreatic cancer, catecholamines activate the β2-AR/HIF-1 axis thus resulting in the production of VEGF that finally triggers neovessel growth (Shan et al., 2013). In the β2-AR/HIF-1 axis, the PKA-dependent phosphorylation of endothelial nitric oxide synthase (eNOS) leads to nitric oxide (NO) production that promotes HIF-1 stabilization and its transcriptional activity in concert with HIF-1 activation either directly by hypoxia or indirectly by the β2-AR pathway, all converging on VEGF production. In embryonic stem cells, for instance, β2-AR antagonism downregulates the eNOS/NO/HIF-1/VEGF pathway thus suggesting that β2-ARs are coupled to a positive feedback loop, which, through VEGFR2 activation by VEGF, leads to vessel growth (Sharifpanah et al., 2014). In endothelial cells (ECs), β2-AR stimulation leads to increased VEGF production and VEGFR2 expression, thus creating an autocrine loop responsible for angiogenic processes (Garg et al., 2017). In this line, in endothelial progenitor cells, β2-AR agonism induces an angiogenic phenotype through the involvement of protein kinase B (AKT) as well as eNOS that, by generating NO, increases VEGF production (Galasso et al., 2013; Ke et al., 2016).

Of the PKA-phosphorylated proteins, the extracellular signal-regulated kinase (ERK) promotes the transcriptional activity of HIF-1 acting through the phosphatidylinositol 3-kinase (PI3K)/AKT pathway. In the OIR model, for instance, β2-ARs have been shown to regulate HIF-1 activity through the involvement of the PI3K/AKT/ERK axis (Su et al., 2023). In this respect, activated AKT, downstream to PI3K, induces the expression of VEGF, which, by interacting with its receptor VEGFR2 reverberates on the PKA pathway either directly or through the PI3K/AKT signaling to ultimately converge on HIF-1 accumulation. In ECs, for instance, β2-AR activation has been shown to be involved in neovessel growth possibly through ERK/AKT signaling (Lemmens et al., 2017).

VEGF leads to the vascularization process through coordinated and synergistic modulation of multiple cell functions, such as EC proliferation and tube formation. In fact, VEGF by interacting with its receptor VEGFR2 on the surface of ECs, triggers EC proliferation, survival and migration. In addition, VEGF expression leads to vascular permeability increase through the production of proteases which allow the extravasation of plasma proteins. In this respect, β2-ARs has been shown to regulate the production and release of matrix metalloproteases (MMPs), which belong to a family of proteases that play a key role in the angiogenic process. (Annabi et al., 2009; Shan et al., 2013; Shen et al., 2018). In fact, basal membrane (BM) degradation by MMPs liberates BM-sequestered growth factors, including VEGF, leading to EC migration and proliferation, tube formation and finally new vessel growth that leads to abnormal glomeruloid vascular plexus with leaky walls and less perfused. In this respect, tip cells, which are present in excessive numbers at the tips of vascular sprouts, are a specialized but transient form of ECs that are characterized by high levels of VEGFR2 (Lee H. W. et al., 2022). In the retina, tip cells at the vascular front sense VEGF gradient and VEGF binding to VEGFR2 results in the activation of specific genes that play a central role in the sprouting growth toward the avascular area (Mühleder et al., 2021). Tip cells guide adjacent stalk cells to determine the function and the morphology of sprouting blood vessels (Kretschmer et al., 2021). After noradrenergic overstimulation, β2-AR switches its coupling to G_i_ thus reversing the G_s_-mediated generation of cAMP and activating alternative signaling cascades including the ERK1/2 pathway (Daaka et al., 1997). If one considers that activated ERK signaling leads to eNOS uncoupling, overstimulation of the β2-AR/Giα/p-ERK pathway mediates reactive oxygen species accumulation and reduces NO bioavailability thus triggering endothelial dysfunction indicating that eNOS uncoupling may have an important role in the pathogenesis of vascular dysfunction by altering endothelial homeostasis (Davel et al., 2014). In addition, reduced PKA activity after β2-AR coupling to G_i_ blunts β1-AR signaling therefore providing a negative feed-forward mechanism to sharpen the transient response of β1-AR to NE upon sympathetic excitation (Yang et al., 2019). No major effects on vascularization process depend on the activation of β1-ARs except for their role in mediating the vasodilation effects of endogenous catecholamines.

**FIGURE 2:**
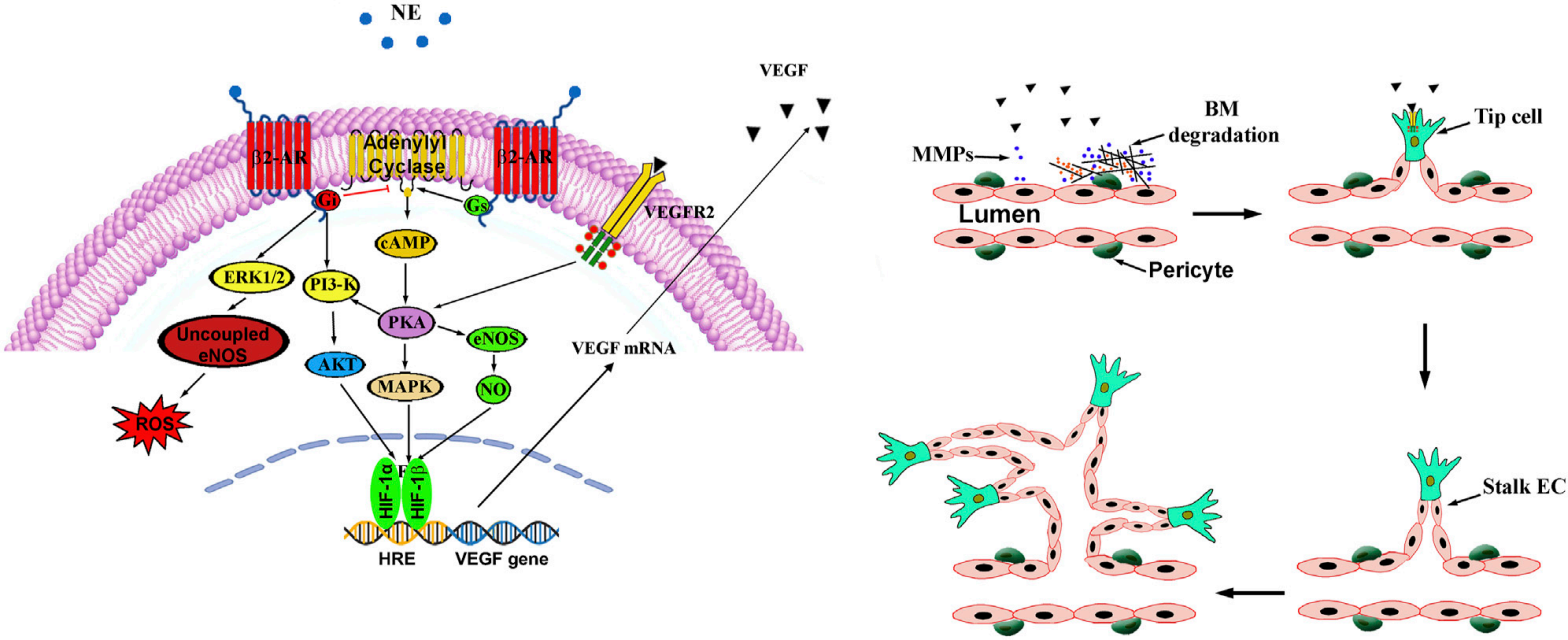
β2-ARs couple dually to Gs and Gi proteins both converging on adenylyl cyclase. In the Gs pathway, increased adenylyl cyclase activity results in elevated cAMP that activates PKA, which, in turn, phosphorylates a number of intracellular proteins that converge on HIF-1 that transactivates a number of proangiogenic genes including VEGF. The final HIF-1-associated player VEGF activates its receptor VEGFR2 to then reverberate on the PKA pathway either directly or through the PI3K/Akt signaling to ultimately converge on HIF-1. Sympathetic overstimulation activates the β2-AR-Gi signaling pathway that counteracts the Gs-mediated generation of cAMP. Gi-associated alternative signalling cascade includes the PI3-K/Akt pathway that regulates HIF-1 expression while the ERK1/2 signaling mediates ROS accumulation by uncoupling eNOS. In this process, stable vessels consisting of aligned ECs connected by tight junctions and covered by pericytes, undergo a cascade of events leading to tight junction loss and protease production, which allows proteins extravasation through BM degradation by MMPs. Pericyte-EC contacts are then lost leading in turn to the release of growth factors that allow vessel sprouting followed by proliferation and migration of ECs that assemble to form the lumen of the neovessel. Pericytes are then recruited, and BM is established. AKT, protein kinase B; β2-AR, β2-adrenergic receptor; BM, basal membrane; EC, endothelial cell; eNOS, endothelial nitric oxidase; ERK1/2, extracellular-regulated kinase 1/2; Gi, inhibitory G-protein; Gs, stimulatory G-protein; MAPK, mitogen-activated protein kinase; MMPs, matrix metalloproteases; NO, nitric oxide; PI3K, phosphatidyl-inositol 3-kinase; PKA, protein kinase A; VEGF, vascular endothelial growth factor; VEGFR2, vascular endothelial growth factor receptor 2; ROS, reactive oxygen species.

In Figure 2, intracellular pathways activated by β2-AR are represented together with a schematic depiction of the main steps leading to vessel proliferation.

### β2-AR in the retina

Low oxygen tension is sensed by chemosensors in the carotid body, which are grouped in clusters of cells each of them containing O_2_-sensing cells, the glomus cells. They are innervated by afferent fibers from the petrosal ganglion and are closely connected with a dense network of fenestrated capillaries. In response to hypoxia, glomus cells activate afferent fibers that project to autonomic centers to induce sympathetic activation (Sobrino et al., 2020).

Sympathetic innervation to the eye originates from the intermediolateral cell column from which descending preganglionic axons reach the superior cervical ganglion (SCG) located posteriorly to the carotid artery, and anterior to the C1-4 vertebrae. From SCG, postganglionic fibers are directed to the choroidal vessels where they release NE that controls blood flow (Wu et al., 2022).

**FIGURE 3:**
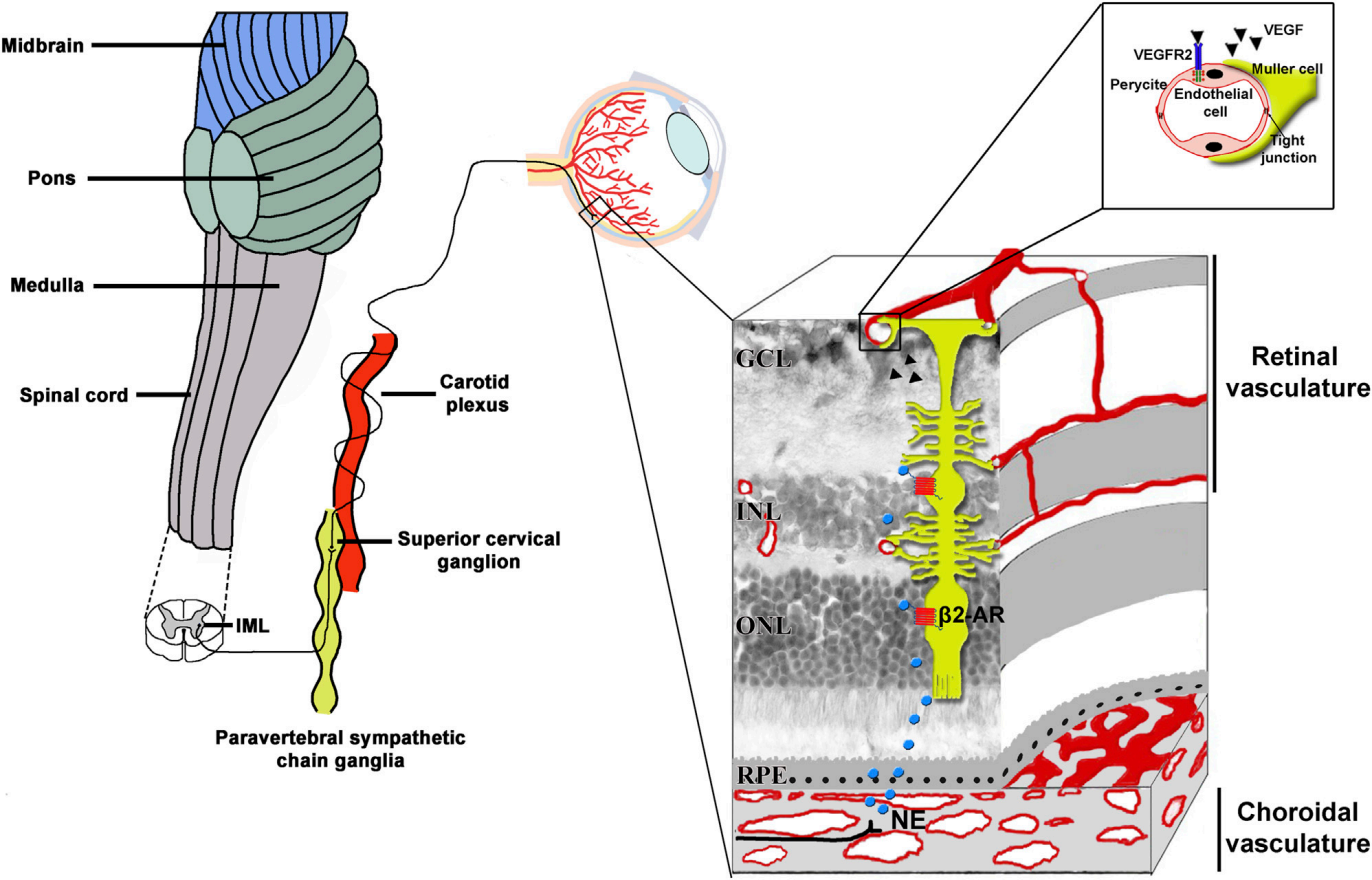
Scheme of the sympathetic innervation of the eye including a sectional view of the layered retinal structure. The sympathetic nerve fibers originate in the IML located in the C8-T2 segments of the spinal cord. From IML, preganglionic fibers activate the superior cervical ganglion cells located in the paravertebral sympathetic chain ganglia, posteriorly to the carotid plexus. From the superior cervical ganglion cells, postganglionic fibers release NE on choroidal vessels from which it reaches the neuroretina by paracrine diffusion. Activated β2-ARs expressed by Müller glia, which spans the entire retinal thickness, trigger a cascade of events which regulates the expression of potent proangiogenic cytokines. Of them, VEGF binds to VEGFR2 expressed by endothelial cells to then activate the process of angiogenesis. In the high magnification, Müller cells endfeet are shown to envelope capillary endothelial cells. β2-ARs, beta2-adrenoceptors; GCL, ganglion cell layer; IML, intermediolateral cell column; INL, inner nuclear layer; NE, norepinephrine; ONL; outer nuclear layer; VEGF, vascular endothelial growth factor; VEGFR2, vascular endothelial growth factor receptor 2; RPE, retinal pigment epithelium.

From the choroid, NE reaches the retina by paracrine diffusion where exerts multiple actions through its coupling to distinct β-ARs (Ruan et al., 2020). Of them, β2-AR is mostly located to Müller cells (Walker and Steinle, 2007; Martini et al., 2011), the major VEGF producer in response to pathological conditions (Bai et al., 2009). Figure 3 represents diagrammatically the sympathetic innervation to the eye.

The fact that β2-ARs are expressed by Müller cells suggests that they may play a role in regulating VEGF production by these cells and that pathological angiogenesis, a key change in many hypoxic/ischemic vision-threatening retinal diseases, depends at least in part on β2-AR activity. Although a growing number of studies have demonstrated the β2-AR plays an important role in catecholamine-induced VEGF expression, the definition of its function is complicated by the overlapping signaling pathways downstream β-AR activation and the limited ligand availability as the plasticity of G protein-coupled receptors requires highly selective ligands (Wu et al., 2021).

Improved understanding of the mechanisms underlying the efficacy of β-AR blockade on hypoxia-induced retinal vessel proliferation may help to clarify β2-AR role in pathological angiogenesis. In the OIR model, for instance, propranolol has been shown to prevent retinal vessel proliferation in response to hypoxia through a downregulation of the HIF1α/VEGF axis via the PI3K/AKT/ERK pathway (Su et al., 2023), further supporting that β-AR activation following catecholaminergic overstimulation may promote angiogenesis through increased VEGF release. This is in line with additional findings demonstrating that inhibiting AKT counteracts PI3K-driven vascular malformations associated to angiogenesis thus confirming that blocking AKT signaling may be a promising therapy for proliferative vascular disorders (Kobialka et al., 2022).

In the OIR model, attenuation of retinal angiogenesis by propranolol administration leads to restored BRB breakdown, which prevents excessive fluid extravasation (Ristori et al., 2011), a major cause of visual impairment in ischemic retinopathies (Rudraraju et al., 2020). In addition, propranolol has been shown to act by reinstating the balance between the apoptotic and the autophagic pathways thus preserving retinal cell survival, which is reflected by the restored electroretinographic responses to light flashes as determined by the recovered amplitudes of the electroretinogram waves (Cammalleri et al., 2017). Restored retinal transduction capability is a major effect of propranolol administration as therapeutic interventions capable to prevent or recover severe visual dysfunction consequent to proliferative retinopathies are still limited in the immature retina as in the case of ROP. For instance, patients with DME show profound improvement in the retinal function after anti-VEGF therapy (Yozgat et al., 2021) while in children with anti-VEGF treated ROP, ERG parameters are still significantly impaired (Curran et al., 2023). In this respect, results from the OIR model are less conclusive with still decreased retinal function and persistent changes in the neuroretinal structures after anti-VEGF treatment although new vessel growth has been delayed (Tokunaga et al., 2014). At variance, recovering retinal vascularization with propranolol may add further efficacy to prevent retinal dysfunction.

**FIGURE 4:**
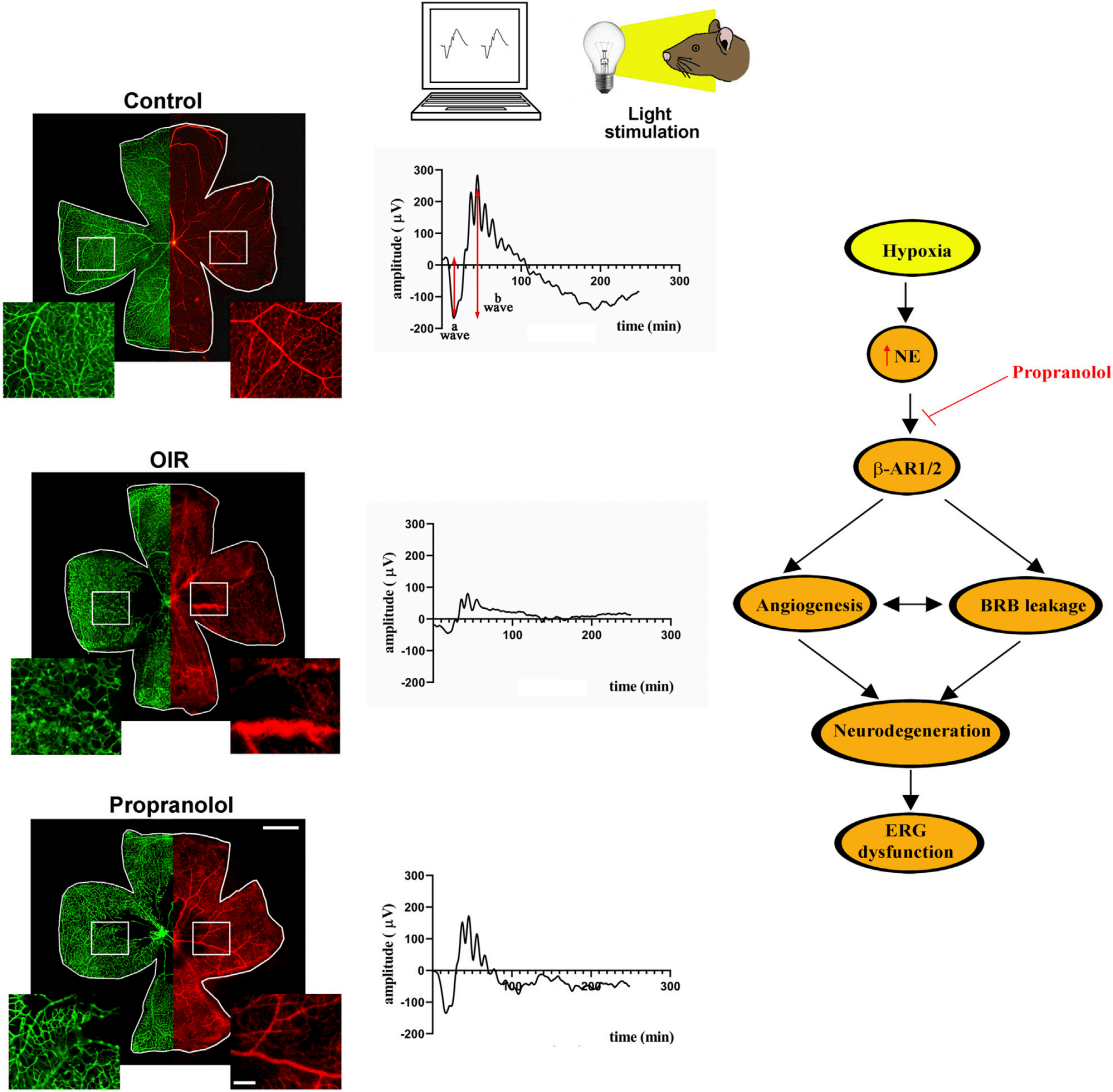
In the OIR model, hyperoxia from PD7 to PD12 leads to capillary obliteration in the central retina that becomes avascular. Following hyperoxia, exposure to environmental oxygen sensed as hypoxia until PD17 leads to vessel proliferation in the midperipheral retina and to BRB breakdown. Systemic administration of propranolol over the hypoxic phase (PD12 to PD17) prevents abnormal vessel growth and recovers BRB breakdown. The representative images of retinal whole mounts refer to IB4 stained retinal vessels with their enlarged magnification (green) and to Evans Blue perfused BRB with its enlarged magnification (red) in the insets. Propranolol treatment counteracts ERG dysfunction to partially recover the amplitudes of the a and the b waves in response to light stimulation. The schematic diagram depicts the role of β-AR blockade in the OIR model. In response to hypoxia, NE released by the sympathetic system activates a series of events that lead to retinal dysfunction. Propranolol administration prevents OIR-associated angiogenic processes thus hampering retinal cell damage and counteracting ERG dysfunction. Scale bar 1 mm in the whole mounts and 200 µm in the insets. BRB, blood retinal barrier; ERG, electroretinogram; IB4, isolectin B4; NE, norepinephrine; OIR, oxygen-induced retinopathy; PD, postnatal day.

In the schematic diagram of Figure 4, propranolol administration to the OIR model is shown to prevent retinal vessel proliferation and BRB breakdown up to restoring electroretinographic responses to light flashes.

### Clinical trials using propranolol in ROP

From early preclinical studies demonstrating propranolol efficacy in the OIR model, the step was short to verify whether in clinical trials, propranolol could be used as an effective therapy in infants with ROP as recently reviewed by Pascarella et al. (2024). In particular, a pilot clinical trial by Filippi et al. (2013) suggested that the administration of oral propranolol (0.25 or 0.5 mg/kg every 6 h) is effective in counteracting the progression of ROP, but it can cause serious side effects. In fact, while in stable infants the treatment with propranolol was well tolerated, serious side effects were observed in infants with unstable clinical conditions who showed cardiorespiratory complications such as bradycardia, bradi/apnea, and hypotension (Filippi et al., 2013). Additional findings demonstrated that oral propranolol reduced ROP progression and the need for junctional treatments (Bancalari et al., 2016) with the further advantage that it could be used to prevent the progression of prethreshold retinopathy to severe ROP (Stritzke et al., 2019). The efficacy of precocious treatment with propranolol was demonstrated by various meta-analyses, the most recent of which has been published in 2024 (Shafique et al., 2024). However, the safety of oral drug administration remains a serious concern. In order to improve the safety profile of propranolol treatment, eye drops have been tested in ROP infants on the basis of preclinical findings demonstrating that in the OIR model topical propranolol reaches the posterior part of the eye (Dal Monte et al., 2013) in which propranolol concentration appears to be much higher than in the plasma as demonstrated in the rabbit eye (Padrini et al., 2014). According to the efficacy of topical formulations of propranolol for IH (Pahl and McLean, 2022), treating infants with propranolol eye micro-drops at a concentration of 0.2% has been shown to reduce ROP progression without side effects (Filippi et al., 2019; Scaramuzzo et al., 2023), while propranolol concentration at 0.1% had no efficacy (Filippi et al., 2017). However, clinical trials using eye drops with propranolol are strongly limited by the low number of preterm infants in the treated cohort and the lack of randomized controlled group to draw definitive conclusions, Although the promising results of the clinical trials might encourage the development of additional studies, determination of the clinical importance of propranolol therapy in ROP requires a more rational approach to the design of prospective clinical trials. On the other hand, there are very few available options to treat ROP and these treatments have limitations. In fact, laser therapy may cause myopia and unfavorable ocular outcome, whereas anti-VEGF therapies may have long-term systemic effects on other organs. Despite the enormous amount of putative drugs tested at the preclinical level, potential pharmacological therapies with partially proven results include caffeine, polyunsaturated fatty acids and vitamin A (Ryu, 2022). In particular, the administration of caffeine was found to reduce ROP progression in a clinical trial (Schmidt et al., 2007) and meta-analysis (Park et al., 2015). In addition, intake of polyunsaturated fatty acids improved visual acuity in infants and reduced the risk of severe ROP (Qawasmi et al., 2013; Hellström et al., 2021). Moreover, vitamin A was found to decrease the progression and incidence of ROP in various clinical trials and a meta-analysis (Darlow and Graham, 2002; Garofoli et al., 2020; Sun et al., 2020).

### Repurposing propranolol for neovascular diseases of the retina

Despite the growing interest on propranolol efficacy in ROP, limited and contradictory information are available on its effectiveness in additional retinal diseases that are characterized by pathological vessel proliferation. Even, some association between β-blockers as anti-hypertensive agents and incidence/progression of nAMD has been suggested (Kolomeyer et al., 2019). Preliminary studies in rodent models of CNV revealed that β-AR blockade with propranolol inhibits CNV progression by reduced expression of VEGF (Lavine et al., 2013; Martinez-Camarillo et al., 2022). This is in line with Montero et al. (2013) who reported that β-blocker therapy is correlated with reduced frequency of the anti-VEGF administration in nAMD patients despite the limited sample size and lack of a control group. On the other hand, Thomas et al. (2015) failed to demonstrate propranolol efficacy in neovascular AMD. In addition, Traband et al. (2017) found that the use of oral β-blockers is not associated with a decreased number of intravitreal injections in nAMD patients. Nevertheless, a synergistic efficacy of anti-VEGF treatment and propranolol has been recently demonstrated in nAMD patients although the major limitations of the clinical trial in which a combination of intravitreal bevacizumab and propranolol has excluded adverse events or signs of ocular toxicity indicating the need of further studies (Silva Tavares Neto et al., 2023). As to additional proliferative retinopathies, oral propranolol did not influence retinal neovascularization associated to PDR suggesting that the anti-VEGF activity of β-AR blockade is too insubstantial to be revealed at the clinical level (Hendrick et al., 2018). On the other hand, propranolol combination with fenofibrate, a medication used to treat abnormal blood lipid levels and adopted to slow down the progression of DR, is more clinically efficient than fenofibrate alone in the treatment of subretinal fluid accumulation causing localized retinal detachment (Shao et al., 2017). In this respect, fenofibrate would likely interact with propranolol by increasing its blood levels and efficacy.

## Conclusion

Despite the myriads of preclinical findings on potential drug candidates for neovascular diseases of the retina, the main therapies used for retinal angiogenesis are still focused on anti-VEGF ligands intraocularly administered. Although anti-VEGF therapies can successfully counteract neovascularization progression, much work has been performed to target unmet clinical needs in the treatment of neovascular pathologies of the retina. In fact, not all patients respond optimally to anti-VEGF therapy, some of them may become refractory over time or may exhibit adverse responses likely associated with repeated intraocular administration. In this complex scenario, targeting the β-AR system in the OIR model has emerged as a promising case of preclinical-clinical translation although limited to clinical trials in ROP. In fact, ROP, at variance with additional neovascular diseases of the retina, includes two well separated phases. The first ischemic phase is characterized by hyperoxia-induced VEGF drop that, in turn, induces the second hypoxic phase characterized by VEGF accumulation that leads to tumultuous vascularization. By reducing VEGF, propranolol administration should be limited to the hypoxic proliferative phase since a further VEGF reduction over the ischemic phase would accentuate ischemia and enhance the subsequent vascular proliferation. In proliferative retinopathies other than ROP, the separation between the two phases is not well defined and the same retina may include ischemic areas, at risk of worsening with propranolol, and proliferative areas, probably benefited by propranolol. In this respect, definition of appropriate time windows in which propranolol could efficiently block retinal vessel proliferation might help to exploit β2-AR as a possible therapeutic target for counteracting pathological angiogenesis in the retina.

Among promising avenues to pharmacologically intervene on neovascular retinopathies, there are several indications about the potential value of systemic therapies inhibiting the overactivation of the sympathetic system in response to an ischemic insult. Further preclinical and clinical testing, drug formulation as well as mechanistic studies aimed to better understanding the role of the β-AR system in OIR would allow to design a reliable therapy that might offer a better efficacy to counteract neovascularization in the eye.
